# Investigation of process parameters for modeling of ceramic composite SiSiC IN dry EDM (DEDM) cutting

**DOI:** 10.1016/j.heliyon.2024.e36459

**Published:** 2024-08-21

**Authors:** Hassan Farooq, Riffat Asim Pasha

**Affiliations:** Mechanical Engineering Deptt, University of Engineering & Technology, Taxila, Pakistan

**Keywords:** Dry EDM cutting, MRR, Taguchi method, Green manufacturing

## Abstract

The manufacturing industry is currently grappling with issues related to energy dissipation and product quality, both of which significantly impact productivity. In addressing this pertinent concern, this study endeavours to identify the key indicators that contribute a crucial role in machining process. The Dry EDM (DEDM) emerges as a novel technology, wherein environmentally friendly gases serve as an alternative dielectric medium instead of liquid EDM approaches. In conventional EDM process, hydrocarbon oils release toxic emissions while the gases used in Dry EDM process don’t produce harmful emissions and hence make the DEDM process sustainable.

An investigation has been carried out to observe the influence of different gases like O2, N2 and Air on Siliconized Silicon-Carbide plate (SiSiC) of ø50mm*2 mm under Dry Electrical Discharge Machining. Effect of different tools is also observed using varying shapes of electrodes like plane Cu electrode of ø6 mm diameter with 60 mm length and tubular shape with outer diameter ø6mm and inner diameter ø5.1 mm with a length of 60 mm. The three gases are one by one supplied through a nozzle in the presence of plane Cu electrode to make the holes in SiSiC plate. The same process is executed with tubular Cu electrode. Modified Taguchi Orthogonal Array is applied to analyze the effect of control factors such as gap voltage, discharge current and pulse on-time through a series of experiments. Effect of different shape of tools and different gases is observed for the material removal rate (MRR) and surface roughness (SR) of specimen.

In this paper, a latest DEDM process of swirl assisted flushing is proposed which provides a new technique to solve the problems of low MRR and poor surface integrity. Swirl assisted flushing works on Decay Law and also microscopic investigation justifies its validity. It is concluded by this research that current and Pulse on time are more contributing factors about 53.3 % and 27 % respectively. Furthermore, it is noted that DEDM increases MRR 19.5 % and lowers Ra approximately 2/3 than traditional machining. The MRR of O2 is about 3.1 times more than Air and N2 comes at least position while N2 creates better quality of surface due to formation of an inert nitride layer. Empirical relations are established for SR and MRR using Minitab 18 Software to develop a robust model. Confirmation test is performed to check the validity of developed mathematical model. The Novelty of this research is also extended by introducing a new method SAF. The results are finally evaluated by ANOVA.

## 1 Introduction

In conventional machining like drilling, milling, boring and sawing there is a direct contact between tool and work piece which leads to high wear and tear. So the quality and surface finish of machined part severely affected. These machining processes are not cost effective and cause low production as discussed by N. Vincent et al. [1].Hence Electrical discharge machining process is introduced in which no direct contact of tool-work piece takes place. In EDM process different types of liquid/oils are used as dielectric media which assist the machining process. The EDM is used for fabrication of complex shapes and also for those materials which are difficult to cut [2]. The hard and tough materials which are not Machine-able by conventional methods, could easily be machined using EDM because there is no direct tool-work piece interface and thus there is less energy dissipation and chances of tool damage decreases up to 70 % [3]. The erosion process is carried out due to the initiation of spark which creates a much higher temperature about 8000-12000ᵒC. Although this high temperature is beneficial for melting and cutting of material yet the too high heat produced during this process causes the deformation of micro structure of the materials, proliferation of thermal stresses and crack propagation [4]. This research gap became an important objective for researchers and hence work started on it to find out the influence of different shapes of tools and different kinds of dielectrics that create pleasant effects on cutting material at high temperature. Secondly to achieve better surface integrity and higher MRR at given circumstances was another objective of this research.

The different types of oils/liquids are used as dielectric medium which assist in machining process. However, these liquid hydrocarbons produce carbon mono oxide and carcinogenic compounds which make this process non-sustainable. Generally speaking, it is concluded that liquid EDM process provides poor dimensional accuracy, low MRR and less removal of debris accumulation [5]. To cope these challenges and to fill out the research gap, the concept of Dry EDM was introduced and became famous now a day in manufacturing industry [6].

Although there are many green liquid dielectrics like Jatropha, neem oil and light Naphthenic oil etc. but in this research dry gases are primarily focused due to their advantages over liquid dielectric as discussed in Table 1. However, Dry electric discharge machining is a novel technology and evolution started in this continuous developing technique since a decade ago. In this innovative technique different gases are used and machining process is carried out in the absence of dielectric liquids. Efforts have been made to understand the phenomenon of erosion by gases without using liquid dielectric.

**Table 1:** Comparative analysis of DEDM and Liquid EDM and benefits of Dry EDM over liquid EDM.

Sr. No	Green Liquid EDM	Green Dry EDM (DEDM)
**1**	A relatively costly technique in which some precious liquids are used like Jatropha and light Naphthenic oil etc [1].	**Cost effective;** it reduces dielectric cost of liquid and related arrangements [7].
**2**	Low performance characteristics i.e. high tool wear and residual thermal stresses are found [2].	**Improvement in performance characteristics;** low tool wear, low residual thermal stresses and higher precision [6].
**3**	Electrolysis takes place due to presence of water/liquid dielectrics that leads to corrosion and surface roughness [8].	**No electrolysis takes place;** hence good agreement with surface finish [7].
**4**	The flushing by liquids is slow and debris are not properly removed which leads to poor surface finish, craters formation and formation of buildup edges (BUE) on tool [9].	**Swirl assisted flushing** (it works on Decay Law and high pressure gases quickly removes the debris and creates better surface finish [10].
**5**	Green liquids such as Neem, Jatropha and canola etc. don’t discharge any toxic emissions [11].	**Environment conscious;** No health hazardous emissions observed by green gases [3].
**6**	Erosion takes place in the presence of dielectric liquid which is very reactive and don’t create pleasant effect on surface integrity [12].	**Inert layer formation;** it is found that by using Nitrogen gas an inert nitride layer formed on the surface of material which produces positive effect on surface integrity [13].
**7**	The hydrolysis produces excess H2 which also creates troubleshoots [14].	**No hydrolysis takes place;** and due to the absence of hydrolysis the unessential gaseous products are not produced [15].

The emerging concept of Dry EDM was first time introduced by the NASA in 1985 where Y. Kimoto et al., used the different gases instead of liquid Dielectric and also explored the advantages of DEDM over conventional EDM process. The researchers successfully tested this emerging method in creating the holes in complex shapes and drilling is carried out through dry EDM process by utilizing the tubular copper electrode with argon and helium gas as dielectric medium. Hence foundation of dry EDM was laid down. Dry EDM is featured by extreme simplicity [16], low tool wear [9], less inter-electrode gap to attain better accuracy [17], least wear of the electrodes due to small gravitational forces of gases [18], and less concentration of heated zone that generates minimum white layer thickness.

Song, D., Liu, K. et al. [19],experimentally explored that ordinary manufacturing process is failed to produce the required product due to the deformation of tool and development of built up edges. Hence during cutting process the tool is worn out which is non-economical [20]. In this context the DEDM method is supposed to be an efficient process to machine hard materials [12] because when the spark propagates, the molten and evaporated specimen is speedily replenished by gaseous dielectric [10].

U. Umer et al. [11],conducted a detailed research and investigated the effects of EDM cutting on microstructure of Alumina ceramics; furthermore, S. Jovic and N. Arsic [8]also experimentally found the wear and tear of electrode during the EDM machining. It is evaluated that doping of TiN or ZrO2 to various ceramic matrixes with 3:1 ratio, dramatically enhances the mechanical properties and conductivity of material increases up to 31 % such as Si3N4–TiN and SiZrO2. The other behavior like oxidation and shock resistance of ceramic materials is explored by E.,Uhlmann, A.,Bergmann et al., [21]. Kong, L., Liu, Z. et al., investigated on Titanium alloys and they carried out parametric variations experimentally to map out the most contributing factors in machining of SiC, SiN/TiN and B4C [22]. It is found that brittle and hard phase in these composite materials primarily create problems during machining operations [7]. Lin, Y.-C. et al.,have evaluated a technique for optimization of high-speed electrical discharge machining (EDM) that significantly influences on EDM machining [14].

The continuous development process in the field of DEDM was carried out by researchers and latest trends emphasis on utilizing the cryogenically cooled gases for better results. Sampath, B., and Myilsamy, used cryogenically cooled oxygen in Near Dry wire cut EDM to investigate white layer thickness [13].In previous researches mostly experiments were focused on steel work piece and copper electrode but recent development concentrated on composite materials of varying geometries of work pieces, tools and dielectrics. Advanced engineering materials are very famous and attracting the attentions in manufacturing industries since last decades and considered as vital element in the modern mechanical systems due to their extra ordinary mechanical and chemical properties [23]. The ceramic materials have unique application because of their high strength [15]and hardness at high temperature [24], oxidation resistance [25]and thermal shock resistance [26].

However, these excellent properties also create difficulties in machining and fabrication of this material which is a major problem. To investigate this rational problem, a ceramic composite Siliconized Silicon Carbid (SiSiC)is used in this research work. The SiSiC utilized in manufacturing industries in wear resistant applications, level sensor and other very hard machining components so these composites are not easy to machine by conventional machining techniques [27]and it is also found that very high temperature generated during machining of SiSiC composite causes the surface damage, cracks proliferation and poor surface finish hence product quality is compromised.

To address these problems this research is conducted to present a dry EDM process with varying geometries of tool electrodes using different dielectric mediums. Plan and tubular electrodes are provided along with supply of different dielectric mediums. It is investigated that easy escape of gases and quick removal of particles lead to the better machining and accuracy.

This research also discusses the effect of different gases on the performance characteristics of dry EDM process and hence to analyze the role of process parameters in productivity. Air, Oxygen and Nitrogen is used in this study as these gases are easily available and abundant in nature (air consists of 78 % N2 and 21 % O2). Table 1. Shows the benefits of DEDM over liquid EDM and it also reflect that DEDM is a green-technology.

**Fig. 1:**
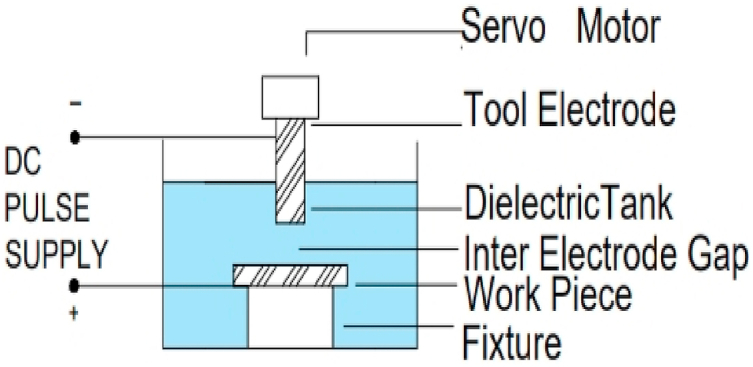
Working principal of die sinker EDM

EDM machine is unique in its working and functionality. The working principal of EDM die sinker is shown in Fig. 1.

## 2 Materials and methods

The experimental plan was developed to conduct the experiments using different green gases (like O2, N2 and Air) as dielectric medium. The siliconized silicon carbide (SiSiC) plate of ø 50 mm with 2 mm thickness was prepared. The SiSiC plate mounted on the fixture and exposed to gases that impinges on the surface of sample and these gases act as dielectric media. The chips and suspended particles are also flush away by the jet of gases. The control panel of EDM machine is programmed according to the set parameters. A gap of almost 0.5 mm was maintained between tool and work piece interface so that the debris may easily be removed and chips accumulation may be avoided.

In this research work, the experiments were performed using a Taiwan made NEUAR-M50 model EDM. The electrode of different shapes like plane and tubular was used. The plane electrode consisting of Copper rod of 60 mm length and ø5mm diameter while tubular electrode with outer diameter ø6mm and inner ø 5.1 mm with a length of 60 mm. Mitutoyo surface measurement tester SJ-210 was used to measure the surface roughness of specimen. Taguchi orthogonal array was implemented to select the process parameters and as well as their levels which are reflected in Table 2.

**Table 2:** Designed Process Parameters (Ip, Ton and V) at their levels.

Sr. No	Factor	Parameter	Units	Level 1	Level 2	Level 3
1	**Ip(A)**	Peak Current	(A)	3	6	9
2	**Ton (us)**	Pulse On Time	(us)	6	8	10
3	**V**	Gap Voltage	(V)	50	100	150

### 2.1 Experimental work

**Fig. 2:**
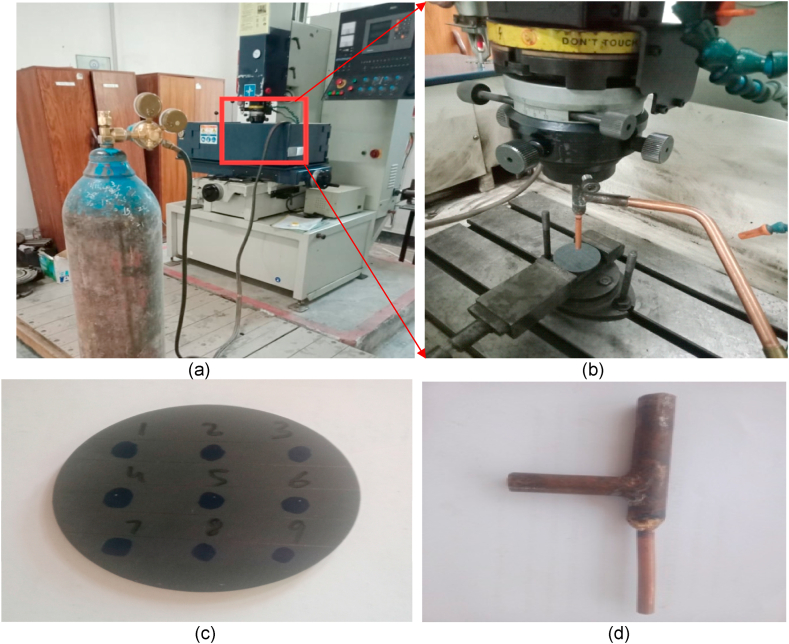
(a)Experimental setup (b) Copper electrode mounted on EDM (c)Prepared specimen SiSiC plate (d) Fabricated Cu tool.

In this experimental work, die sinker machine is used to machine the hole of 6 mm diameter in work piece. The work piece is circular disc shape of SiSiC with diameter 50 mm and 2 mm thickness as shown in Fig. 2 composition of SiSiC composite is given below in Table 3a, Table 3b.

**Table 3a:** Chemical composition of work piece SiSiC.

Sr No.	Composition of SiSiC					
**1**	Si	SiC	C	Fe2O3	SiO2	Al2O3
**2**	0.98 %	93.9 %	Trace	Trace	4.1%	0.3%

**Table 3b:** Material properties of SiSiC.

Properties	values	Properties	values	Properties	values
Hardness	HV11 ≥ 24 GPa	Density	3.05–3.14 g/cm3	Linear Expansion Coefficient	4.2x10-6/K at 25–395 °C
Thermal Conductivity	122 to 205 W/mK	Thermal Shock Resistance	ΔT 826 ᵒC	Young’s Modulus	370 to 420 MPa

The series of experiments were performed on EDM (NEUAR-M50) with plane and tubular copper electrode and detail of experimental set up is mentioned below in Fig. 2(a–d). Dry EDM operation is performed using various gasses (like Air, Oxygen and Nitrogen).

In this study the control panel is set according to the desired machining plan and the path is given by selecting the axis like x-axis, y-axis and z-axis respectively. When the tool path is fixed and all parameters are set then the servo motor is actuated to drill the holes according to the program. After completion of each machining operation, one tool is dismantled while another is mounted to carry out next operation. The experimental conditions are mentioned in Table 4.

**Table 4:** Experimental conditions in EDM.

Work piece	SiSiC Disc (dia; 50 mm)
Electrode Material	Copper
Dielectrics	Air, Oxygen, Nitrogen
Current(A)	3, 6 and 9
Voltage(V)	50, 100, 150
Pulse On Time(us)	6, 8, 10

**Fig. 3:**
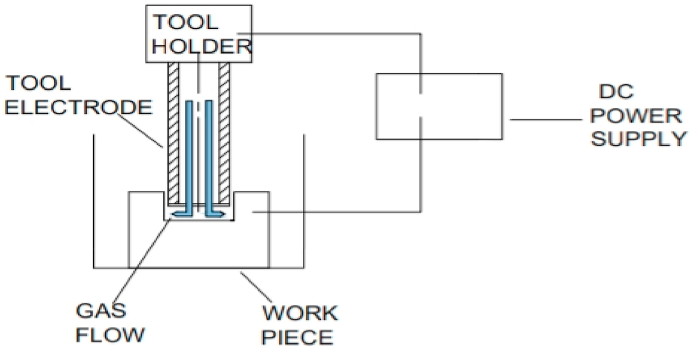
Schematic of Dry EDM process.

The schematic diagram is shown below in Fig. 3.

### 2.2 Experimental design

There are multiple parameters that effect on response variables, out of which the three main factors Ip, V and Ton are selected that plays major role in quality of surface texture and MRR [3]. Taguchi Orthogonal Array L9 is used to design the experimentation for drilling of specimen SiSiC Plate using Cu electrode and gases as dielectric medium. The 3 different levels of peak current (3A, 6A and 9 A), pulse on time (6us, 8us and 10 us) and gap voltage (50v, 100v and150v) are selected as shown in Table 5.

**Table 5:** Design of Experiment using Taguchi (L9) orthogonal array.

SR.#	Taguchi OA (L9)			Tool shape	1^st^ Round	2^nd^ Round	3^rd^ Round
	Peak Current (Ip), A	Pulse On Time (Ton), us	Gap Voltage (v), V				
1	3	6	50	Plane	Air	Nitrogen	Oxygen
2	6	8	100	Plane	Air	Nitrogen	Oxygen
3	9	10	150	Plane	Air	Nitrogen	Oxygen
4	3	6	100	Plane	Air	Nitrogen	Oxygen
5	6	8	150	Plane	Air	Nitrogen	Oxygen
6	9	10	50	Plane	Air	Nitrogen	Oxygen
7	3	6	150	Plane	Air	Nitrogen	Oxygen
8	6	8	50	Plane	Air	Nitrogen	Oxygen
9	9	10	100	Plane	Air	Nitrogen	Oxygen

In this Table # 5, Nine no. of experiments were performed using only Air in 1^st^ round, then in 2^nd^ round 9 experiments were performed but another gas Nitrogen is supplied and in 3^rd^ round only Oxygen is used. So these 27 experiments were performed with different gases but in presence of same shape of Plane-Cu tool (1^st^ phase). Similarly, 27 experiments were again repeated in 2^nd^ phase using Tubular-Cu tool with Air, N2 and O2 respectively. Hence total **54** experiments were performed to analyze and evaluate the effect of different gases and effect of shape of tools on output responses. The detail of these 54 experiments was described in summarized form in Table 8, Table 9 respectively. In the last portion, novelty of this research is also extended by introducing a new method SAF.

**Table:** Nomenclature

SiSiC	Siliconized Silicon Carbide
DEDM	Dry Electrical Discharge Machining
MRR	Material Removal Rate(mm3/min)
OA	Orthogonal Array
SR	Surface Roughness (um)
Pon	Pulse on Time (us)
Ip	Peak Current (A)
SAF	Swirl Assisted Flushing
SN Ratio	Signal to Noise Ratio

### 2.3 S/N ratio optimization in taguchi approach

In the Taguchi method, the S/N ratio basically implies the optimal effect of the set parameters/factors. There are three cases which are usually considered in optimizing techniques like maximal target values, minimal target values and the nominal target values. In this study the “Minimal Ra” and “Maximal MRR” are our target values. The corresponding equations for nominal, max and min are discussed below.

(a) Nominal is Better:

In this case there is no need to minimize objective function or maximize it but nominal is considered as Best as shown in equation 2.1

(2.1) $$\frac{S}{N}=10\;\log\left(\frac{\overset{⃐}{y}}{s_{y}^{2}}\right)$$

(b) Larger is Better:

In this case there is a need to maximize the objective function (e.g. Material Removal Rate) and equation (2.2) is used for larger the better case.

(2.2) $$\frac{S}{N}=-10\;\log\left(\frac{1}{n}\sum_{i=1}^{n}\frac{1}{y_{i}^{2}}\right)$$

(c) Smaller is Better:

In this case there is a need to minimize the objective function (e.g. Surface Roughness) and equation (2.3) is used for smaller the better.

(2.3) $$\frac{S}{N}=-10\;\log\left(\frac{1}{n}\sum_{i=1}^{n}y_{i}^{2}\right)$$

### 2.4 Experimental procedure

#### 2.4.1 Tools of different shapes

Different shapes of copper electrodes were used to investigate the effect of their shape on response variables. Two common types of tools that prominently influences on output parameters are as fellows.

(1) Plane-Cu tool (2) Tubular-Cu tool.

#### 2.4.2 Different types of dielectric

The studies show that there is prime role of dielectric in Dry EDM machining because it directly influences on the quality of surface finish and MRR as well. In recent experimental work, different types of dielectric fluids are used as under:

(1) Oxygen Gas (2) Nitrogen Gas (3) Air.

In Dry Electrical Discharge machining (DEDM), an alternative dielectric in gaseous state is used instead of liquid hence the sensitivity of conventional method is changed. The properties of these gases including Oxygen, Air and Nitrogen are mentioned in Table 6.

**Table 6:** Electrical, mechanical and thermal properties of gases (dielectric media).

Sr. No	Properties	Gases		
		O2	Air	Nitrogen
**1**	Dielectric strength (MV/m)	2.6	3.0	2.8
**2**	Dielectric constant	1.00049	1.000536	1.00058
**3**	Dynamic velocity (g/m s)	0.020	0.019	0.017
**4**	Thermal conductivity (W/m K)	0.026	0.026	0.0254

### 2.5 Using Plane-Cu electrode and dielectric gases (O2, N2 and air)

A. (i) Put the SiSiC specimen in fixture after cutting into the disc shape.
(ii) Mount the Cu electrode on the EDM.
(iii) Weight of specimen was calculated before machining.
(iv) Run the machine according to the suitable program as instructed to CNC computer and select control parameters.
(v) Initiate machining operation using Plane-Cu electrode and Air as Dielectric.
(vi) After completion of machining operation work piece was again weighted.
(vii) The same process was repeated by changing controlling parameters with same tool and dielectric gas.
B. Repeat the same process again using same Plane-Cu tool with N2 gas.
C. Repeat the same process using Plane-Cu tool and O2 gas.

**Fig. 4:**
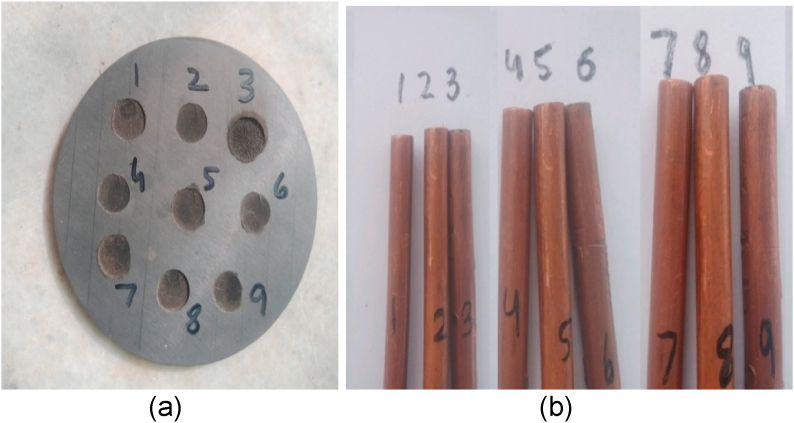
(a) Holes on work piece (b) Plane copper electrodes.

The machined SiSiC plate and Plane-Cu tool is shown in Fig. 4(a and b). The average erosion time of these gases was between 1 and 5 min and the depth of hole is approximately 0.5 mm to 1 mm depends upon the flushing gas. Gas parameters are mentioned in Table 7.

**Table 7:** Gas parameters for experimentation.

Sr. No.	Gas Parameters	Units	Numerical value
1	Flushing pressure	Bar	20 bar
2	Erosion time	Min	5 min
3	Depth of hole	Mm	0.5–1.0 mm
4	Flow rate	ml/min	10 ml/min
5	Temperature	°C	Room temp
6	Electrode polarization	–	Negative

**Table 8:** Taguchi OA and machining performance measure using Plane-Cu electrode.

Sr. No	Peak Current Ip(A)	Pulse On Time Ton(us)	Gap Voltage (V)	Air		Nitrogen		Oxygen	
				MRR (mm3/min)	Ra (um)	MRR (mm3/min)	Ra (um)	MRR (mm3/min)	Ra (um)
1	3	6	50	0.0411	1.357	0.0172	1.214	0.1302	2.152
2	6	8	100	0.0512	3.751	0.0234	3.321	0.8112	4.670
3	9	10	150	0.0431	5.415	0.1644	6.365	0.1925	6.423
4	3	6	100	0.0454	2.665	0.0338	2.412	0.5321	3.331
5	6	8	150	0.0521	3.648	0.0204	2.852	0.8121	3.648
6	9	10	50	0.0514	4.719	0.0205	4.415	0.8223	4.229
7	3	6	150	0.0585	2.852	0.0181	3.358	0.7272	2.665
8	6	8	50	0.0845	3.331	0.0208	3.789	0.6912	3.751
9	9	10	100	0.0433	4.980	0.0613	4.874	0.4344	3.257

**Table 9:** Taguchi OA and machining performance measure using Tubular-Cu electrode.

Sr. #	Peak Current Ip(A)	Pulse On Time Ton (us)	Gap Voltage (V)	Air		Nitrogen		Oxygen	
				MRR (mm3/min)	Ra (um)	MRR (mm3/min)	Ra (um)	MRR (mm3/min)	Ra (um)
1	3	6	50	0.0085	1.672	0.0116	2.350	0.0212	1.213
2	6	8	100	0.0243	1.320	0.0268	2.322	0.0158	1.451
3	9	10	150	0.0698	4.312	0.0244	4.943	0.9001	2.012
4	3	6	100	0.0196	1.999	0.0131	1.820	0.0234	1.080
5	6	8	150	0.0148	3.005	0.1042	2.471	0.0155	2.011
6	9	10	50	0.0266	1.103	0.0333	1.675	0.0195	3.820
7	3	6	150	0.0580	3.704	0.1532	1.022	0.0255	1.855
8	6	8	50	0.0151	1.951	0.0491	1.992	0.0244	1.348
9	9	10	100	0.0141	2.010	0.0128	1.661	0.0299	2.437

Taguchi Methodology was executed which is combination of statistical and mathematical analysis. The MRR and SR values of specimen were obtained under the influence of Air, N2 and O2 respectively at designed parameters using Taguchi orthogonal array as shown in Table 8.

### 2.6 Using Tubular-Cu electrode and dielectric gases (O2, N2 and air)

**Fig. 5:**
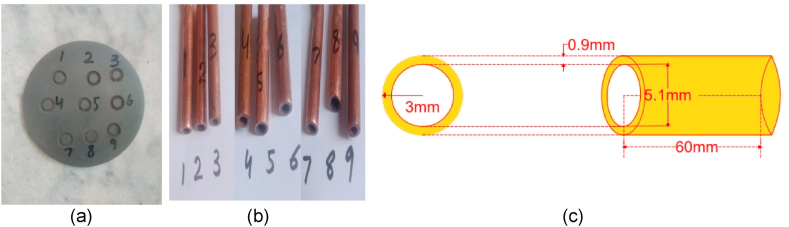
(a)Machined work piece (b) Tubular electrodes used (c) Cross-section of tubular tool

In this session, tubular copper electrode of outer diameter ø 6 mm and inner ø5.1 mm is used as shown in Fig. 5(b and c) and supply of gases was provided through the tube of electrode to drill the holes in specimen as described in Fig. 5 (a). The detail of experimental procedure is mentioned below:

**A.** (i) Put the SiSiC specimen in fixture after cutting into the disc shape. (ii) Mount the Cu electrode on the EDM (iii) weight of specimen was calculated before machining (iv) Run the machine according to the suitable program as instructed to CNC computer and select control parameters (v) Initiate machining operation using Tubular-Cu electrode and Air as dielectric (vi) after completion of machining operation work piece was again weighted.

B. Repeat the same process again using same Tubular-Cu tool with N2 gas.
C. Repeat the same process using Tubular-Cu tool and O2 gas.

In this experimental setup, 9 experiments were performed using Air then N2 and finally O2, hence total 27 experiments were performed and evaluated using software Minitab to analyze the effect of different process parameters (Ip, Ton and V) on the output responses like MRR and Ra etc. The Material Removal Rate (MRR) can be determined using the following Formula in Equation (2.6)

(2.6) $$\mathrm{MRR}\;\left(mm3/\min\right)=\frac{Wtb-Wta}{T^{*}\rho }\;$$

Wb: weight of work piece before machining.

Wa: weight of work piece after machining

t: machining time (min). ρ: density of the material (g/cm3)

The experiments conducted on different level of peak current (L1 = 3,L2 = 6, L3 = 9 A);

Pulse on time (L1 = 6,L2 = 8, L3 = 10 us);

Gap voltage (L1 = 50,L2 = 100, L3 = 150 v)

## 3 Results and discussion

The results were analyzed by using design expert 9.0 software. Series of experiments were performed and these results were evaluated by Analysis of Variance. It is prominent in the summary provided by ANOVA table regarding the vast range of results like regression model, P-values, lack of fit, Means and standard deviations obtained during experimental tests.

### 3.1 Material removal rate and surface roughness

The MRR after each experiment is calculated by given equation (2.6). It is mentioned here that the experimental output responses were analyzed by using the software Minitab 19. All factors that influences on the Dry EDM cutting of ceramic composite SiSiC are analyzed. It is very clear from Table 10, mentioned below that the MRR and Surface Roughness are significantly affected by controlling factors. The P-value 0.0001 clearly indicating the significance of the model and significance of other parameters as well.

**Table 10:** ANOVA table for material removal rate.

Factors	DOF	Sum of Squares	Mean Squares	F-value	P-value
Model		0.32	0.11	1255.23	<0.0001 significant
Peak Current	2	0.17	0.26	1583.65	<0.0001
Pulse on time	2	0.15	0.22	1331.33	<0.0001
Gap Voltage	2	0.51	0.14	8640.15	<0.0008
Error	2	4.11	1.86	1.514	0.5881
Total	8	4.234			
S = 1.29 R-sq = 95.9 % R-sq(adj) = 91 %					

#### 3.1.1 Using plane tool with air

**Fig. 6:**
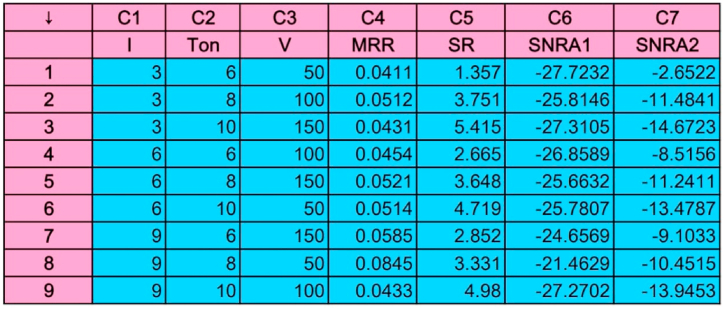
Software Analysis of machining parameters and response variable.

**Fig. 7:**
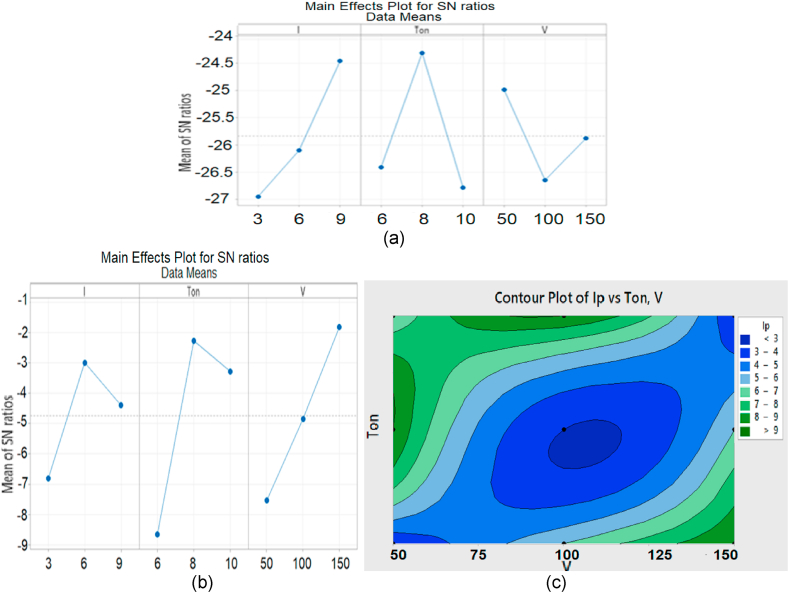
S/N Ratio graphs using Air and plan tool (a) MRR (b) Ra (c) Contour plot of Ip vs Ton, V.

The blast of Air is ejected through nozzle at high pressure (20 bars) and this high pressure air not only flushes the debris but also facilitates the sparking process. Hence this constant spark causes the better erosion. The Software Analysis using Plane tool and Air is represented in Fig. 6. Column C6 and C7 shows the S/N ratios corresponding to MRR and Ra (C4 and C5) respectively. It is evident from graph (in Fig. 7) that minimum MRR is observed by the Ip at its 1^st^ level (3 A) while the pulse on time at 2^nd^ level (8 us) causes maximum MRR. The jet of high pressure air continuously removes the metal chips which support the MRR process rigorously. It is also investigated that the high kinetic energy molecules of Air become fully charged when these molecules are allowed to expand suddenly through nozzle. Hence these charged particles play a very crucial role in making dielectric film which supports the sparking process.

The Ra values for each experiment are obtained from the surface roughness tester and it is found that minimum Ra is generated by Ton at its 3^rd^level (10 us). Similarly, Response graph for Means values and Response graph for Signal to Noise ratios also shown and evaluated in Fig. 7(a–c).

#### 3.1.2 Plane tool with N2 gas

**Fig. 8:**
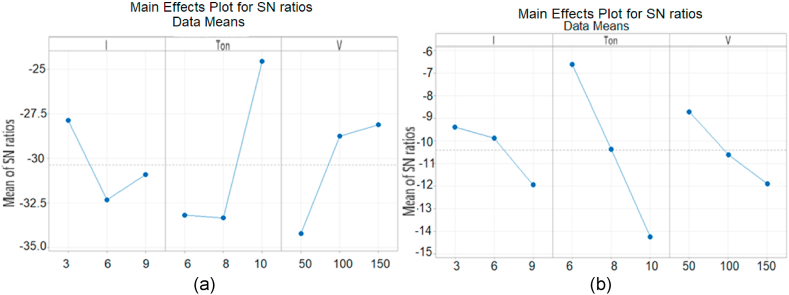
Means effect plots and S/N Ratio for N2 (a) MRR (b) Ra (c) 3D scatter Graph of Ip vs Ton vs V for N2.

Plane tool is used along with Nitrogen gas to find out the effect of Nitrogen gas on MRR and Ra of material which is represented in Fig. 8(a and b). It is found that the values of MRR and Ra are influenced by Ton mainly (at 10 us and 6 us respectively). It is evident from graph that maximum MRR is created by Ton. Similarly, it is also found that minimum Ra is generated by Ton at its 3^rd^ level (10 us). Current (3 A) also plays its role while the participation of third process parameter that is gap voltage (150v) remained comparatively less significant for MRR and Ra respectively.

The Nitrogen gas is naturally a non-reactive gas and its inert behavior makes it different from other elements of the same group in periodic table.

Nitrogen gas superficially generates a nitrite layer on the surface of specimen and this layer creates a very pleasant effect and enhances the surface finish.

#### 3.1.3 Plane tool with O2 gas

**Fig. 9:**
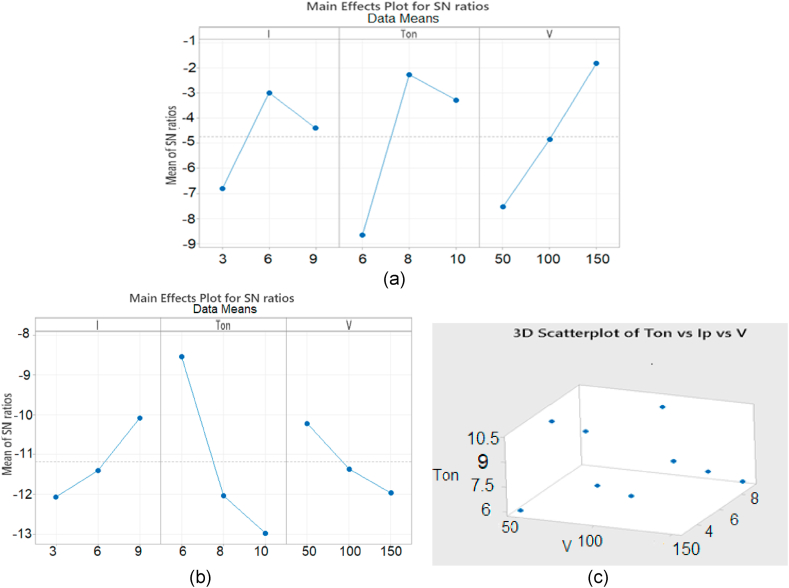
Means effect plot and S/N Ratio for O2 (a) MRR (b) Ra.

Software Analysis using Plane tool and O2 is represented in Fig. 9(a–c). It is found that the 3^rd^ level of gap voltage (150 V) contributes in generating maximum MRR however there is no significance contribution of gap voltage in Ra and by increasing the values of gap voltage there is exponentially decrease in Ra values. However, minimum value of surface roughness is obtained at the 3^rd^ level of process parameter Ton (10 us). Current is not playing a significant role in this case. The maximum MRR of specimen is noticed due to the oxidizing behavior of Oxygen and this oxidizing property facilitates the burning process which leads to the better erosion. Hence high MRR is observed.

#### 3.1.4 Tubular tool with air

**Fig. 10:**
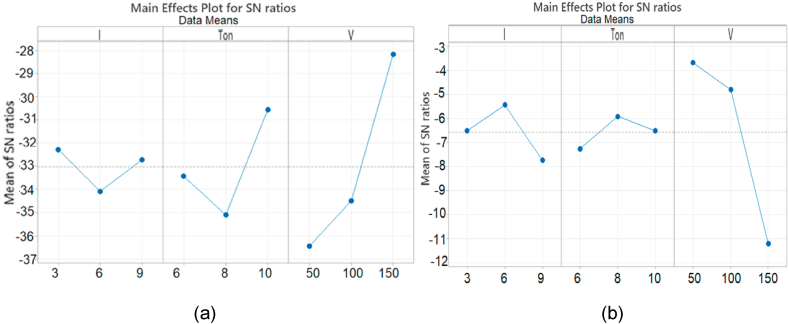
Means effect plot and S/N Ratio for Air (a) MRR (b) Ra.

In this experiment Air is ejected through the tubular tool electrode at high pressure and the tool is designed in such a way that the pressurized gases produce a strong swirl according to Decay Law. This swirl movement with high kinetic energy assists in flushing the chips and suspended particles are quickly replenished. Software Analysis using Tubular tool and Air is represented in Fig. 10(a and b).

It is found that the values of MRR and Ra are mainly influenced by gap voltage. It is evident from graph that maximum MRR is created by gap voltage. It is also found that minimum Ra is generated by V at its 3^rd^ level (150 us) while the participation of Ip is seemed to be suppressed here in this case. The purpose of swirl movement is to enhance the flushing and also maintain a strong electro-static charge of dielectric for sparking process to get maximum erosion and MRR.

#### 3.1.5 Tubular tool with nitrogen gas

**Fig. 11:**
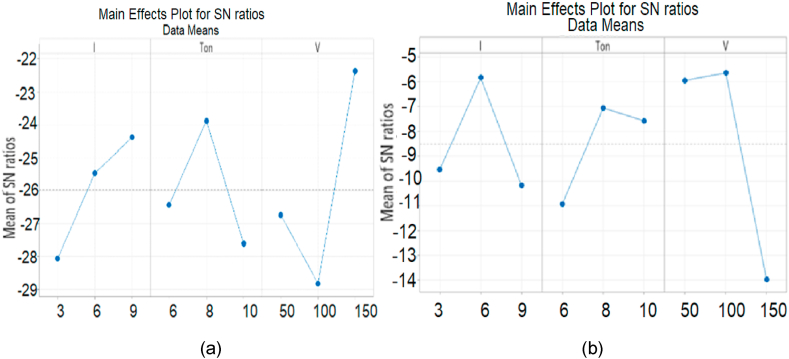
Means effect plot and S/N Ratio for N2 (a) MRR (b) Ra.

Experiments were planned using Nitrogen gas to establish the role of dielectric medium. Nitrogen gas is used at standard temperature to find out the effect on MRR and SR respectively using tubular tool. It is evident from graph that the current is least contributing factor on MRR while gap voltage is maximum influencing parameter. On the other hand, minimum surface roughness is obtained by 3^rd^ level of gap voltage (150v). It is observed from graph that maximum MRR is created by gap voltage (150 V) followed by Ton (8 us). Similarly, it is also noticed that minimum Ra is generated by V at its 3^rd^ level (150 V) followed by the Ton (6us) as shown in Fig. 11(a and b).

#### 3.1.6 Tubular tool with Oxygen Gas

**Fig. 12:**
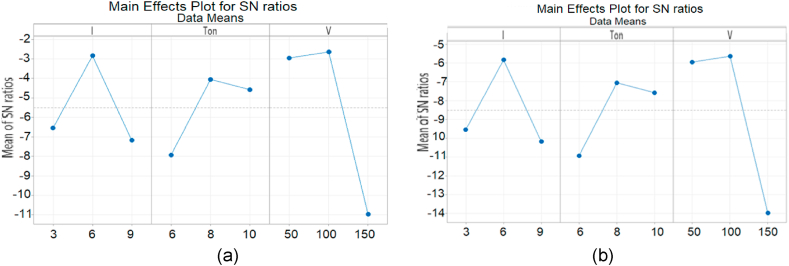
Means effect plot and S/N Ratio for O2 (a) MRR (b) Ra.

Oxygen gas is used to find out the effect on MRR and Ra respectively while a tubular Cu-tool of 6 mm outer diameter and 5.1 mm inner diameter is mounted on EDM. It is evident from graph that maximum MRR is found on Ton at 1^st^ level (6us), Similarly the voltage is affecting on Ra and minimum Ra is observed at 3^rd^ level (150 V), followed by pulse on time (6 us). The current (6A) has also plays its role on Ra as shown in Fig. 12(a and b).

Oxygen gas supports the sparking process and ultimately stronger spark produces high temperature. This high temperature facilitates the erosion process.

### 3.2 ANOVA

Analysis of variance test was performed to check the performance measure and analyze the role of contributing factors. ANOVA was done at α = 0.10 (significance level) i.e. 90 % confidence level. Also those factors have significant contribution to performance measure having P-value< 0.0001. It is evident that the P-value of peak current is (0.0001) and Ip is significant process parameter similarly p-value of Ton showed its significance. Although gap voltage is also a contributing factor on performance measure with less influence. ANOVA results are mentioned in Table 10.

After performing ANOVA test, the values of different factors are obtained which reflected that the peak current (Ip), voltage (V) and pulse-on time (Ton) are the significant terms that impart major role in determining the fate of MRR model. It is also noted that Lack of fit F-value proved that it is insignificant and thus, there is little chance that “Lack of fit value” could happen due to the noise. It is also prominent that the “R2” value of 0.95 agreed reasonably with the “Adj R-Squared” value of 0.91 as discussed in ANOVA Table 11.

**Table 11:** ANOVA table for surface roughness.

Factors	DOF	Sum of Squares	Mean Squares	F-value	P-value p-value > F
Model		3.89	0.81	5262.23	<0.0001 significant
Peak Current	2	0.71	0.71	2073.66	<0.0001
Pulse on time	2	0.34	0.34	1105.33	<0.0001
Gap Voltage	2	0.31	0.31	2485.46	<0.0001
Lack of Fit	20	3.113E-005	7.7112E-006	3.11	<0.092 not significant
Error	8	1.67E-003	1.9E-004		
S = 2.31 R-sq = 99 % R-sq(adj) = 99 %					

It is found that the R-square values are very important and in this case the R-square vales are very close to 100 % which reflect that the variations are controlled. It is also a good explanation of the model that the values of R-square and adjusted R-square are found to be above 95 % which proves that this model shows a relation between input parameters and output responses. It is evaluated that much higher value of MRR was measured in DEDM process with comparatively low tool wear and build up edges (BUE) when compared with liquid EDM.

It is investigated that P-value of Ip and Ton are less than 0.10 which justifies that Ip and Ton has significant effect on Response variable and ascending order of contribution of these parameters is the Ip ≥ Ton ≥ V respectively. After performing ANOVA, relationship of factors by multiple linear regressions is obtained and mathematical model is obtained.

(5.1) MRR = 2.25 + 0.74*A+0.160*B-0.25*C-0.0170*A*B-0.320*A31.011*B2 -0.17*C2

(5.2) Ra = 3.81 + 0.5*A+0.80*B-0.0290*C+0.084*B*C-0.19*A32.11* B

Where V is gap voltage, Ton is pulse on time and Ip is the peak current. It is noticed that the three parameters are undoubtedly significant but out of these three process parameters, Ip is most contributing factor to the response. Optimal values of control factors (i.e Ip, Ton and V) achieved from Taguchi method then the values of the aforementioned factors are put into the multiple linear equations (5.1), (5.2)). it is also found that Results obtained from ANOVA closely match with the results obtained from Taguchi method.

It is also found that the MRR increases considerably with the increase of current. Similarly, when the pulse on time raises it also cause the marginally increase in MRR. Basically when current increases then the heat energy rate increases and this extra amount of heat energy ultimately facilitates the level of MRR. Low MRR occurs at low Ip which also shows that the low V and low Ton provide good surface finish. The statistically analysis of ANOVA proved that the models which are being developed, can predict within 90 % of confidence level of values of MRR and Ra. Despite of it, there are the regression models which obtained during ANOVA analysis and R2 values. The R2 values were found to be 95.5 % for MRR, 99 % for Ra. It was found that the experimental results approximately similar to the predicted values.

ANOVA also predicts the optimum parameters that were obtained by multiple response optimizations. The process parameters which significantly play their role in determining the fate of output response variables at optimum values are achieved and their optimum values are 3A peak current, 8μs pulse-on time and 150 V gap voltage. To confirm the validity of the models, series of experiments were performed with the optimized parameters in multiple response optimizations.

### 3.3 Effect of discharge current on material removal rate

It is also observed that the discharge current is a very significant parameter and it greatly influences on Material Removal Rate. That is why the confidence level of current is highest than all other contributing factors mentioned above. As indicated in the main effects plot there is sharp increase in MRR by increase in current. So it is investigated that an increase in current causes an increase in flushing process leading to higher MRR. The contribution of current in removing MRR is found 53.3 % compared to other factors.

### 3.4 Effect of pulse-on time on material removal rate

It is found that the Statistical analysis of MRR revealed that the effect of pulse-on time (Ton) on MRR is also significant and it influences the process at 95 % confidence level. It is observed that there is a major role of pulse-on time which is about 27 % because it contributes to the pulse energy which has vital role in removing the small chips and enhances the mechanism of MRR. Increased pulse on time creates enough energy to facilitate the Material Removal Rate.

### 3.5 Effect of voltage on MRR

As the results obtained from statistical analysis using ANOVA, the gap voltage doesn’t play significant role in determining the fate of how much Material is removed in experiments using different shapes of electrodes. The confidence level of voltage is lower than all other contributing factors which rules out its minor contribution in determining the fate of MRR. It is known that voltage enhances sparking action. A comparison of parameter’s effect on MRR in these experiments is presented in ANOVA Table.

This table gives how the importance of processing parameters changes when the electrodes are changed. The relative importance of processing parameters changes significantly with the use of tubular electrodes. In the case of plane electrodes, Ton is the highest contributor to MRR followed by current. However, with tubular electrode, current plays major role followed by pulse-on time (Ton).

### 3.6 Comparative analysis of gases

The comparative analysis of different green gases was done to investigate the influence of these gases on the output variables.

#### 3.6.1 Material removal rate

Different flushing gases like Air, Nitrogen and Oxygen were used as dielectric in experimentation and their behavior is observed as shown in Fig. 13A (a,b). It is found that the lowest MRR is measured with molecular N2 as flushing gas while flushing with Air resulted about three times higher values of MRR than N2. It is noted that the highest MRR is measured with oxygen which is 0.9001 mm3/min at process parameters (9A, 10us, 150v) because it serves as oxidizing gas and measured value of MRR with oxygen is about 3.1 times the MRR with air and almost above 9 times than the value with nitrogen. The MRR was observed 21.38 % higher than nitrogen gas which is 0.1925 mm3/min.

The Oxygen has highest MRR than N2 and Air because Oxygen is not only a strong oxidizing agent but also very reactive gas and facilitates the sparking process of EDM; hence stronger spark produces high temperature which causes better erosion. So O2 has higher MRR than Air and N2 (as both these gases are comparatively inert). Furthermore, N2 makes nitrite layer on the surface of specimen which reduces MRR. Air consists of 78 % N2 so the properties of air resemble to N2.

#### 3.6.2 Surface roughness

The surface roughness is very important factor; it demands optimality in industrial products. The value of Ra is discussed in Fig. 13 (A), Fig. 13 (B)B (a,b) which is measured during experimentation using different flushing gases. It is investigated that the better surface finish is obtained using nitrogen gas while the Ra generated using Air has higher value than nitrogen. Similarly, Oxygen played its significant role as higher MRR during erosion and highest value of Ra than nitrogen and Air which is almost 6.423μm at (9A, 10us, 150v). The value of Ra ruled out for oxygen is about three times higher than Air because of strong oxidation behavior. Highest value of Ra 6.423μm is measured for Oxygen at control parameters (9A, 10us, 150v). It is concluded that better surface finish is produced by using N2 gas which is 1.022μm at process parameters (3 A, 6us, 150v). So surface roughness observed by using Oxygen gas is 15.88 % higher than Nitrogen gas.

**Fig. 13 (A):**
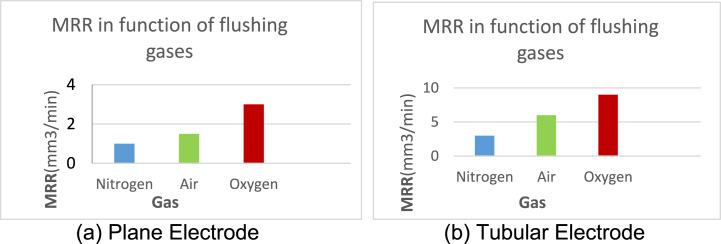
MRR for flushing gases N2, Air and O2.

**Fig. 13 (B):**
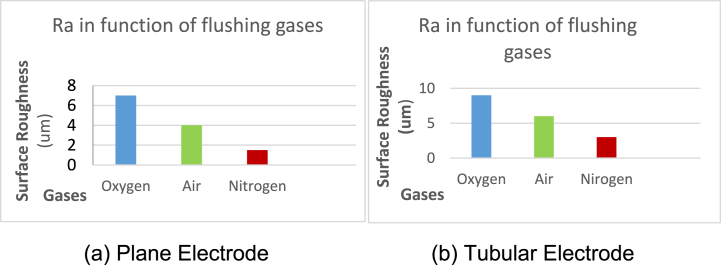
Surface roughness for flushing gases N2, Air and O2.

It is investigated that Nitrogen gives better value of surface roughness (1.022μm) because during the EDM process, N2 forms a superficial layer of nitrite at the surface of specimen and this protective layer declines the roughness process.

### 3.7 Morphological analysis

**Fig. 14:**
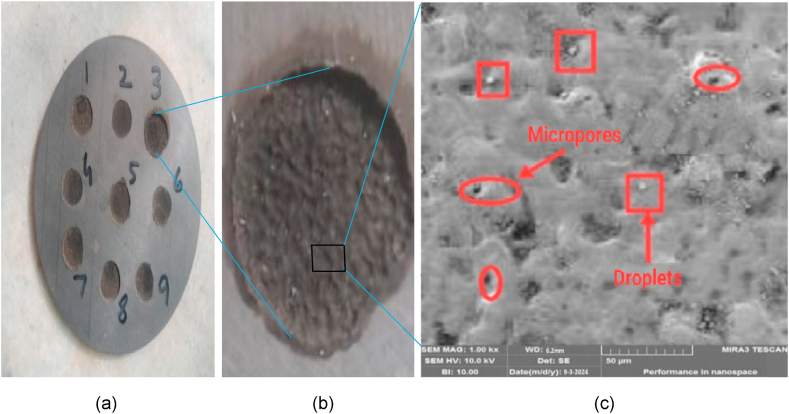
(a)Machined work piece using plane electrode (b) Surface texture of drilled hole(c) Topography at micro level (at Ip = 9A,Ton = 10μm, V = 150 v).

The morphology of machined work piece under SEM is shown in Fig. 14(a–c). It shows the surface texture and the effect of machining parameters on morphology of specimen. On the surface of machined work pieces some cracks, cavities, globules, craters and some suspended particles are found which are responsible for enhancing the surface roughness and lowering the MRR. It is further evaluated that due to the existence of these micro-cracks and cavities the phenomenon of spalling is observed because of excess stress occurred that exceeds beyond the tensile strength of the material. The micro details of work piece and electrode also helps us to understand and establish the results.

#### 3.7.1 Work piece

The surface texture of Siliconized Silicon Carbide was observed under the SEM. It was observed that tool-work piece interface became blunt and having rough surface with pitted scars and craters formation due to thermo-hydrodynamic wear process. High temperature creates the Thermal stresses that ultimately lead to proliferation of micro thermal-cracks and craters. Hence it causes the higher surface roughness of machined work piece. The detailed morphology of work piece is shown in Fig. 14 below which shows the micro cracks, droplets and microspores at Ip = 9A, Ton = 10μm, V = 150 v. High energy produced during machining creates thermal stresses and the stresses beyond the specific limit causes cracks propagation resulting a phenomenon known as spalling. Spalling creates very negative effect on surface morphology of machined work piece. It is investigated that the surface texture of work piece using tubular electrode is more fine than using plane electrode because the area of contact is higher in plane electrode compared to tubular electrode ultimately more heat is produced during machining hence more hydrothermal stresses are produced.

**Fig. 15:**
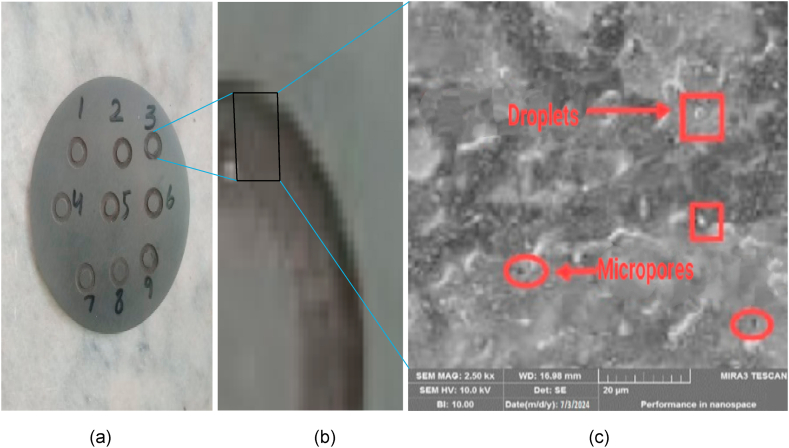
(a) Machined work piece using Tubular Electrode (b) Surface texture of drilled hole (c) Topography at Micro Level (at Ip = 9A, Ton = 10μm, V = 150 v).

Furthermore, a material facing high temperature is more susceptible to deform as compared to lower temperature. Secondly the flushing of debris and suspended particles is better due to swirling mechanism of ejected gases through pipe. Undoubtedly, the micro-cracks are produced during machining of specimen using tubular electrodes but better surface quality is achieved due to quick removal of chips as shown in Fig. 15(a–c).

#### 3.7.2 Tools electrode

**Fig. 16:**
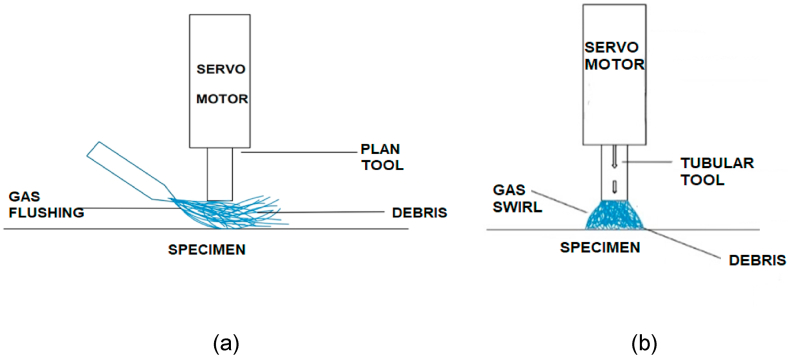
Schematic of tools (a) Direct flushing using plane tool (b) Flushing through tubular tool by Swirl.

In this research work two shapes of tools were used including plane copper electrode and tubular copper electrode with green gases. The electro erosion process conclusively defines the resulting morphology of the machined surface. It is investigated that the higher MRR was obtained using tubular electrode as compared to plane copper electrode because gases passed through the pipe of tubular electrode generating swirl resulted better flushing of debris takes place as compared to the direct impinging of gases on specimen. The swirl of cutting gases produced during Dry EDM cutting of sample is shown in Fig. 16 (b). It is evident from figure that this powerful swirl of gases is produced when high pressure gases are ejected through the electrode pipe and gives better flushing of chips leading to the better surface finish. It is found that after each spark, a reasonable decrease in pressure takes place which causes a small localized explosion hence in a result a molten material is flushed away that forms the surface crater. These craters abruptly enhance the mechanism of surface roughness. The direct impinging of gases on specimen creates comparatively less kinetic energy to remove the chips as shown in Fig. 16 (a).

**Fig. 17:**
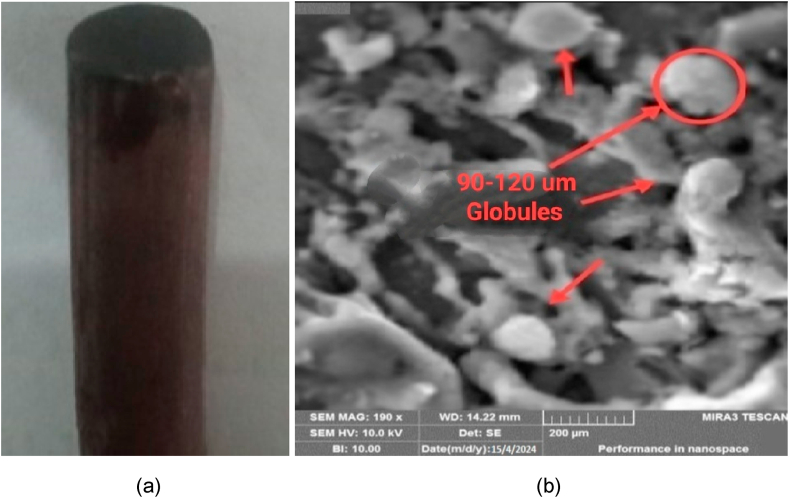
(a) Plane-Cu tool after Erosion(b) Microscopic topography of tool.

**Fig. 18:**
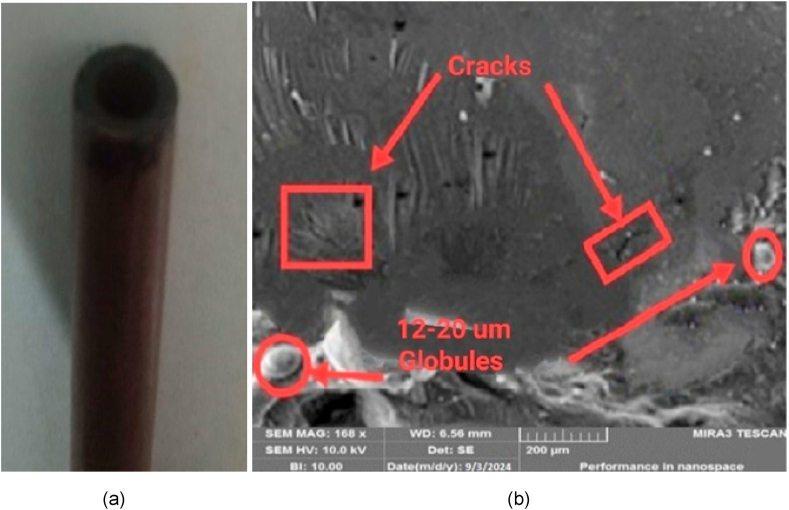
(a) Tubular-Cu tool after Erosion (b) Microscopy at high resolution.

The comparative analysis of micro-details of deformed plane and tubular copper tools are shown in Fig. 17(a and b) and Fig. 18(a and b) respectively. It is observed that the explosion due to high heat in plan tools produces thermal stresses when these stresses propagate beyond the strength limit of material, then the micro-cracks are formed. The microscopy shows that the globule formation is higher in plane tool compared to tubular tools due to the dielectric swirl and spalling phenomena. The sizes of globules found in plane tools are 90–120 μm at (6A, 8us, 50v) while 12–20 μm is measured in tubular tools.

### 3.8 Confirmation test

It is rechecked by confirmation test that either the value obtained is matches with desired results or not and how much percentage error lies there in the variations. So these optimum levels of control parameter are summarized in Table 12. And these parameters are needed to validate the models developed for material removal rate and surface roughness. The confirmation test is performed to validate the developed mathematical models using the optimum parameters which are evaluated by software. The optimum control parameter and response variables are mentioned below with percentage errors.

**Table 12:** Optimum parameters and response variable with (% Error).

Gases	Optimum Parameter Setting			Percentage Error (%)	
	Ip (A)	Pon (us)	Voltage(V)	MRR (mm3/min)	Ra (um)
Air	3	6	50	8.31	2.9
	9	10	150	2.7	1.10
N2	9	10	150	1.12	1.42
	3	6	150	0.56	1.92
O2	9	10	50	7.78	7.8
	6	8	150	1.14	9.2
Average Predicted Error				**3.60**	**4.05**

In this table it is observed that the percentage error calculated for response variables is less than >10 %. The error less than 10 % falls under acceptable limit. Hence the good reproducibility of this experimental research work is confirmed and the mathematical models developed for surface roughness and material removal rate are also validated. Furthermore, this test was helpful to establish the conclusion that the better surface quality and high MRR can be obtained using DEDM process in the presence of green gaseous dielectric.

## 4 Conclusions

The primary goal of this research work was to investigate the effect of different gases and different shape of tools on the MRR and SR to get quality product. Dry EDM process has been evaluated for different process parameters including Ip, Ton and V. Specified results are mentioned as follows.

➢ The results showed that oxygen has 3.1 times more MRR than Air followed by Nitrogen gas. The highest MRR is measured with oxygen which is 0.9001 mm3/min at process parameters (9A, 10us, 150v) while MRR obtained by N2 is (0.0116 mm3/min) which is almost 9 times less than O2. The Oxygen has highest MRR than N2 and Air because Oxygen is not only a strong oxidizing agent but also very reactive gas and facilitates the sparking process of EDM; hence stronger spark produces high temperature which causes better erosion. MRR obtained by O2 is 77.59 % higher than N2 gas.
➢ The microscopic studies showed that the plane tools produce large globules (90–120 μm at 6A, 8us, 50v) as compared to the globules generated by tubular Cu-tool electrodes (12–20 μm at 6A, 8us, 50v). Hence the Ra value for plane tool is increases almost 16.7 % as compared to tubular tool.
➢ It is measured that the Ra value for Air is higher than nitrogen gas while the highest value of Ra is achieved by Oxygen which is almost 6.423 μm at (9A, 10us, 150V). It is concluded that best surface finish is produced by N2 gas which is 1.022 μm at process parameters (3 A, 6us, 150v). So surface roughness observed by using Oxygen gas is 15.88 % higher than Nitrogen gas. Oxygen boosts up spark process of EDM and causes localized explosions which flush away the molten chips and in a result the craters are formed. These craters enhance surface roughness.
➢ It is noted that during experimentation using a plane electrode, an exponential increase in MRR was observed whereas a steady increase in MRR was obtained when tubular tool was used.
➢ In case of MRR it is observed that Ip and Ton are most dominant factor about 53.3 % and 27 % respectively while gap voltage plays minor role in determining the MRR value of work piece.
➢ In this paper a new technique, swirl assisted flushing (SAF) was conceptualized in which high pressure gases ejects through a pipe that causes swirl flushing under Decay Law which gives dramatically better effect on MRR and surface integrity while direct impinges of gases on the surface of work piece using plane electrode is less effective. The MRR observed by swirl assisted mechanism was (0.9001 mm3/min) and on the other hand it was achieved 0.1925 mm3/min by direct impinges using plane tool. Hence MRR by swirl assisted mechanism is 21.38 % higher than direct flushing.
➢ It is found that MRR is 19.5 % higher in Dry EDM process than conventional EDM. This research work can be a reference for the researchers who are EDM users and specifically for those who want to use this rare ceramic composite (SiSiC) work piece in industrial applications.
➢ Gas analyzers are used for detection of harmful gaseous emissions but no harmful and health hazardous emission was found in this study. So it is proved that this experimental work falls under the umbrella of green manufacturing.
➢ The model developed are also validated through confirmation test and hence it is concluded that the Dry EDM machining of SiSiC ceramic material in the presence of different gases like (Air, N2 and O2) have good agreement with higher MRR and lower Ra with percentage error less than 10 %.

### 4.1 Outlook section

Although Dry EDM is a novel technology and it gives better results than conventional EDM process however in this research work a very high temperature was found between inter electrode gap during sparking (about 8000–12000 ᵒC) and it is needed to control this high temperature because it causes thermal stresses, spalling, cracks propagation, and deformation of micro structure of material. In addressing this research gap, the mixing of some mist contents in the gases can give valuable output by controlling and lowering the temperature and hence further improvement in surface finish is possible. For this purpose, the scheme of experimental design would be modified by addition of simply a supply of water with controlled valve and nozzle.

It is investigated that some gases like O2 produces high MRR meanwhile it does not have good effect on surface integrity. Similarly, other gases like N2 generates good surface finish but it has less contribution towards MRR. So it is suggested that mixing of these gases in different ratios/percentages could produce desired results. It is also planned to use the spray of dielectric fluids in future for reducing the cost of dielectric as well as to get the effectiveness of homogenized spray components (atomized contents). Furthermore, some bio-oils are under investigation like esterification of NEEM oil which can be used to achieve better results. There are several shapes of tools that can be extended but in this research, the better results have achieved by using tubular shape so this kind of shapes (like tubular/hollow/pipes/multi-channeled and slotted etc.) should be further tested.

## Data availability statement

The “data will be made available on request.”

## CRediT authorship contribution statement

**Hassan Farooq:** Writing – review & editing, Writing – original draft, Visualization, Validation, Software, Resources, Project administration, Methodology, Investigation, Formal analysis, Data curation, Conceptualization, PROFESSOR. **Riffat Asim Pasha:** Supervision.

## Declaration of competing interest

The authors declare that they have no known competing financial interests or personal relationships that could have appeared to influence the work reported in this paper.
